# Genetic Polymorphisms CYP3A4*22, CYP3A5*3, and CYP2D6 Predicted Phenotypes Are Not Associated with Antipsychotic Treatment Outcomes in Neurotypical Prepubertal Boys with Conduct Disorders

**DOI:** 10.3390/ph19091401

**Published:** 2026-09-04

**Authors:** Dmitriy V. Ivashchenko, Mikhail D. Che, Farid R. Aysin, Svetlana N. Tuchkova, Ivan N. Korsakov, Ekaterina I. Ianavichiute, Mariia A. Ivashchenko, Pavel V. Shimanov, Rimma V. Kondratieva, Artem V. Shubin, Karin B. Mirzaev, Yuriy S. Shevchenko, Dmitry A. Sychev

**Affiliations:** 1 Federal State Budgetary Research Institution, Russian Research Center of Surgery Named After Academician B.V. Petrovsky, Moscow 119435, Russiadmitry.alex.sychev@gmail.com (D.A.S.); 2 General Psychiatry Department, Scientific-Practical Children’s and Adolescents Mental Health Center n.a. G.E. Sukhareva, Moscow 119334, Russia; 3 Research Institute for Molecular and Personalized Medicine, Russian Medical Academy of Continuous Professional Education, Moscow 125993, Russia; 4 Veltischev Research and Clinical Institute for Pediatrics and Pediatric Surgery of the Pirogov Russian National Research Medical University of the Ministry of Health of the Russian Federation, Moscow 117513, Russia; ima.clin.gen@gmail.com; 5 Department of Clinical Pharmacology and Therapeutics, Russian Medical Academy of Continuous Professional Education, Moscow 125993, Russia; 6 Department of Child Psychiatry and Psychotherapy, Russian Medical Academy of Continuous Professional Education, Moscow 125993, Russia

**Keywords:** gene, genome, genetics, genotype, antipsychotics, CYP3A, CYP2D6, conduct disorders, children

## Abstract

**Objectives**. To identify associations between CYP3A4*22 and CYP3A5*3 genotypes, CYP2D6 phenotype, and the effectiveness and safety of antipsychotics in neurotypically developed boys with conduct disorders. **Methods:** The study included neurotypically developed boys aged 7–12 years who were hospitalized for conduct disorders. All patients were prescribed an antipsychotic. Patient follow-up lasted 14 days. Treatment effectiveness was assessed using a clinical aggression assessment (checklist) and the CGI-S, CGI-I, and CGAS scales. Safety was assessed using the UKU SERS and SAS scales. Patients were examined upon enrollment in the study, on day 5, and on day 14. All patients were genotyped for the CYP3A4*22 (rs35599367, C>T), CYP3A5*3 (rs776746, 6986T>C) CYP2D6*3 (rs35742686), CYP2D6*4 (G1846A, rs3892097), CYP2D6*6 (rs5030655), CYP2D6*10 (C100T, rs1065852), CYP2D6*41 (rs28371725) loci. CYP2D6 metabolism type was determined based on genotyping results, and patients were divided into two subgroups: those with normal metabolism (NM) and those with intermediate or poor metabolism (IM + PM). **Results:** Patients taking carbamazepine (*n* = 11) were excluded from the analysis of associations between treatment outcomes and the CYP3A4*22 and CYP3A5*3 polymorphisms. The analysis of associations between treatment outcomes and CYP2D6 metabolism type was conducted in two stages: the overall sample and a subsample of patients who were prescribed risperidone (*n* = 80). No significant associations were found between carrier status of the CYP3A4*22 and CYP3A5*3 polymorphisms and treatment effectiveness parameters. Analysis of the overall sample did not reveal any significant associations between CYP2D6 metabolism subtypes and the effectiveness parameters of drug therapy. Analysis of patients receiving risperidone revealed one statistically significant association: patients with CYP2D6 IM + PM reported headaches more frequently (16.1% vs. 2%; *p* = 0.03). Carrier status of the CYP3A4*22 polymorphism was significantly associated with asthenia and lethargy on day 5 (50% vs. 9.4%; *p* = 0.008). **Conclusions:** Our study identified only a few significant associations between the CYP3A4*22 polymorphism, CYP2D6 slow metabolism, and patients’ reports of early adverse reactions in a 14-day observation period. Further research is needed to identify pharmacogenetic predictors of the efficacy and safety of antipsychotics in neurotypical children with conduct disorders.

## 1. Introduction

Currently, the pharmacogenetics of antipsychotics is quite clearly focused on adult patients with schizophrenia [1]. Most studies include patients over the age of 18 [2,3,4]. There is a lack of research on patients with diagnoses other than schizophrenia, a fact reflected in the guidelines for the personalized selection of antipsychotics [1]. To date, the most extensively studied genes are *CYP2D6* and *CYP3A4* [1]. Other genes are not yet included in the guidelines for personalized selection of antipsychotics.

The *CYP2D6* gene, unlike *CYP3A4* and *CYP3A5*, has been studied in considerable detail. To date, guidelines have been published for the selection of haloperidol, aripiprazole, brexpiprazole, zuclopenthixol, and risperidone based on *CYP2D6* genotyping [1,5]. Risperidone and aripiprazole are the most extensively studied drugs in pharmacogenetic research. There is well-established evidence of a correlation between CYP2D6 metabolism type and the concentration of aripiprazole or risperidone in the blood [3,5,6,7,8]. Cytochrome CYP2D6 is a useful diagnostic target, as its metabolic rate is highly likely to correspond to the results of pharmacogenetic testing [1,5]. To determine the metabolic type, genetic testing is performed for the *CYP2D6* haplotypes CYP2D6*3 (rs35742686), CYP2D6*4 (G1846A, rs3892097), CYP2D6*6 (rs5030655), CYP2D6*10 (C100T, rs1065852), CYP2D6*41 (rs28371725), as well as CYP2D6*1 duplications [1].

Antipsychotics are used in children not only to treat schizophrenia, but, very frequently, to treat conduct disorders. To date, all published pharmacogenetic studies on the treatment of conduct disorders in children have focused on individuals with autism spectrum disorders, and only a few of these include *CYP3A4* and *CYP3A5* genotyping [7,9,10]. However, even assuming that the studies in question measured CYP2D6 metabolism rates, the results cannot be extrapolated to the population of neurotypical children. Neurotypical children were included in pharmacogenetic studies of antipsychotics only as part of mixed cohorts [5]. However, studies focusing on neurotypical children with conduct disorders are also needed for practical application.

The CYP3A4 isoenzyme plays a unique role in the metabolism of xenobiotics: it is involved in the metabolism of more than 50% of drugs [11]. This isoenzyme is encoded by the *CYP3A4* gene, which is highly polymorphic: there are more than 60 known polymorphisms [11]. However, polymorphisms are rarely found in people of European descent [12]. The influence of the CYP3A4*22 rs35599367 (C>T) polymorphism is frequently observed [12]. The carrier status of the CYP3A4*22 polymorphism leads to impaired translation and, consequently, is associated with low CYP3A4 activity in the human liver [12].

An additional complication arises from the related CYP3A5 isoenzyme, which shares more than 80% of its substrates with CYP3A4 [11]. When *CYP3A5* is expressed, this isoenzyme metabolizes both its own substrates and those of CYP3A4. Among Caucasians, homozygous status of the CYP3A5*3 polymorphism (rs776746, 6986T>C) is very common; this variant “shuts down” the expression of the isoenzyme [13]. As a result, CYP3A5 is often in a resting state in Caucasians because CYP3A5*3 (rs776746) inactivates its expression [13]. According to various sources, between 82% and 95% of people of European descent are carriers of the CYP3A5*3 allele [14]. However, the presence of even a single active CYP3A5*1 allele leads to high levels of *CYP3A5* expression in the liver [11]. For example, CYP3A5 expression in the liver of a CYP3A5*3/*3 (GG) carrier may be 4%; if one active allele (CYP3A5*1/*3) is present, expression is 50% [15]. The amino acid sequence of CYP3A4 and CYP3A5 is 84% identical, which ensures that the substrates of these isoenzymes are nearly identical [16,17]. In individuals who are carriers of the CYP3A5*1 allele, more than 80% of CYP3A4/5 substrates are metabolized by the CYP3A5 isoenzyme [11].

Pharmacogenetic testing of *CYP3A4* and *CYP3A5* polymorphisms is widely used to guide tacrolimus prescribing [18,19,20]. There are also studies on the effect of *CYP3A4* and *CYP3A5* polymorphisms on the efficacy of breast cancer treatment [21,22], safety of diazepam [23], alprazolam [24], serum level of midazolam [25], and carbamazepine [26].

In 2024, the *CYP3A4* gene was first included in the Dutch Pharmacogenetics Working Group (DPWG) guidelines on pharmacogenetic testing for quetiapine dose selection [1]. Previous studies have demonstrated that the CYP3A4*22 genotype influences the plasma concentration of quetiapine [27]. Meanwhile, *CYP3A5* genotyping is not included in the DPWG recommendations [1]. However, the similarity between the substrates of the CYP3A4 and CYP3A5 isoenzymes calls for further research to more accurately predict the efficacy and safety of antipsychotics [11]. The CYP3A4 isoenzyme is involved in the metabolism not only of quetiapine, but also of lurasidone, cariprazine, risperidone, aripiprazole, ziprasidone, perphenazine, and haloperidol [28]. Polymorphisms in the *CYP3A4* gene are rarely found among Asians, unlike those in *CYP3A5* [29]. Consequently, studies involving genotyping for both *CYP3A4* and *CYP3A5* are needed. Such studies to clarify the role of *CYP3A5* in the efficacy and safety of quetiapine are already underway, but they have limitations regarding study design and the ethnic background of participants [29,30]. Studies conducted in the Caucasian population have yielded conflicting results regarding the effect of CYP3A4*22 and CYP3A5*3 polymorphisms on plasma quetiapine concentrations [27,31]. Investigating the effect of the CYP3A5*3 polymorphism on the efficacy and safety of other antipsychotics holds promise [32]. However, to date, there have been only a few studies on this topic, and they have involved adult patients [33].

The prevalence of CYP3A4*22 in the Russian population is similar to that in the European population (6.8%) [34]. The prevalence of the CYP3A5*1 (active) allele in Russian patients was found to be 2.27% by Zastrozhin et al. (2017) [33] and 17.6% by Ivashchenko et al. (2017) [35]. Thus, investigating *CYP3A4* and *CYP3A5* polymorphisms as biomarkers of the efficacy and safety of antipsychotics is a pressing task.

There is evidence of differences in the expression of *CYP3A4* and *CYP3A5* genes depending on gender [36,37]. In particular, *CYP3A* expression is higher in women than in men [36]. This is confirmed primarily by laboratory experiments [38], but must be taken into account when planning the clinical study. Therefore, when studying the pharmacogenetics of CYP3A substrates, it is preferable to include patients of the same sex to avoid differences in pharmacokinetics.

We previously conducted a pilot study on the prognostic significance of CYP3A4*22 and CYP3A5*3 for the efficacy and safety of antipsychotics in 84 neurotypical children with conduct disorders [39]. We have since expanded the sample size and genotyped CYP2D6 to obtain more accurate results. The aim of this study was to assess whether CYP3A4*22, CYP3A*5, and the predicted CYP2D6 phenotype are associated with early treatment response and early adverse reactions during the first 14 days of antipsychotic treatment in neurotypical boys with conduct disorders.

## 2. Results

### 2.1. Sample Description

Pharmacogenetic test results were available for 115 patients. Clinical and demographic characteristics are shown in Table 1.

All patients were neurotypical, with no comorbid intellectual disability or autism spectrum disorder; however, Wechsler test results were available for only 77 patients. A diagnosis of “intellectual disability” was ruled out in all patients through clinical evaluation. Carbamazepine was used as a mood stabilizer in 11 patients, valproate in two, and lamotrigine in one case. Antidepressants used: sertraline (*n* = 5), fluvoxamine (*n* = 2), amitriptyline (*n* = 1). Patients who had been prescribed antidepressants or other medications, except for carbamazepine, were not excluded from the analysis.

The subsample after excluding patients with carbamazepine included 8 carriers of the CYP3A4*22 rs35599367 polymorphism (1 homozygous TT), and 14 carriers of CYP3A5*1 rs776746 (of whom 1 was homozygous CC). The frequencies were consistent with the Hardy-Weinberg distribution. No significant differences in demographic parameters were observed between carriers of the CYP3A4*22 and CYP3A5*3 polymorphic and “wild-type” genotypes (Supplementary Table S1).

No significant differences were observed between the different CYP2D6 metabolism types in the overall sample. In the subgroup of patients prescribed risperidone, those with IM + PM were significantly younger (9 [8; 10] vs. 10 [9; 11] years; *p* = 0.048) and shorter (1.42 vs. 1.45 m; *p* = 0.045) compared to NM, but their body mass index did not differ (Supplementary Table S2). An analysis of pharmacotherapy (Table 2) revealed no significant differences in daily antipsychotic dosages or in the prescription of adjunctive pharmacotherapy.

### 2.2. Analysis of Treatment Effectiveness Based on CYP3A4*22 and CYP3A5*3 Genotypes

The effectiveness of pharmacotherapy for conduct disorders, as assessed by the CGAS, CGI-S, and CGI-I scales, did not differ significantly based on carrier status of CYP3A4*22 and CYP3A5*3 polymorphisms. The results of the analysis are shown in Supplementary Table S3.

**Figure 1:**
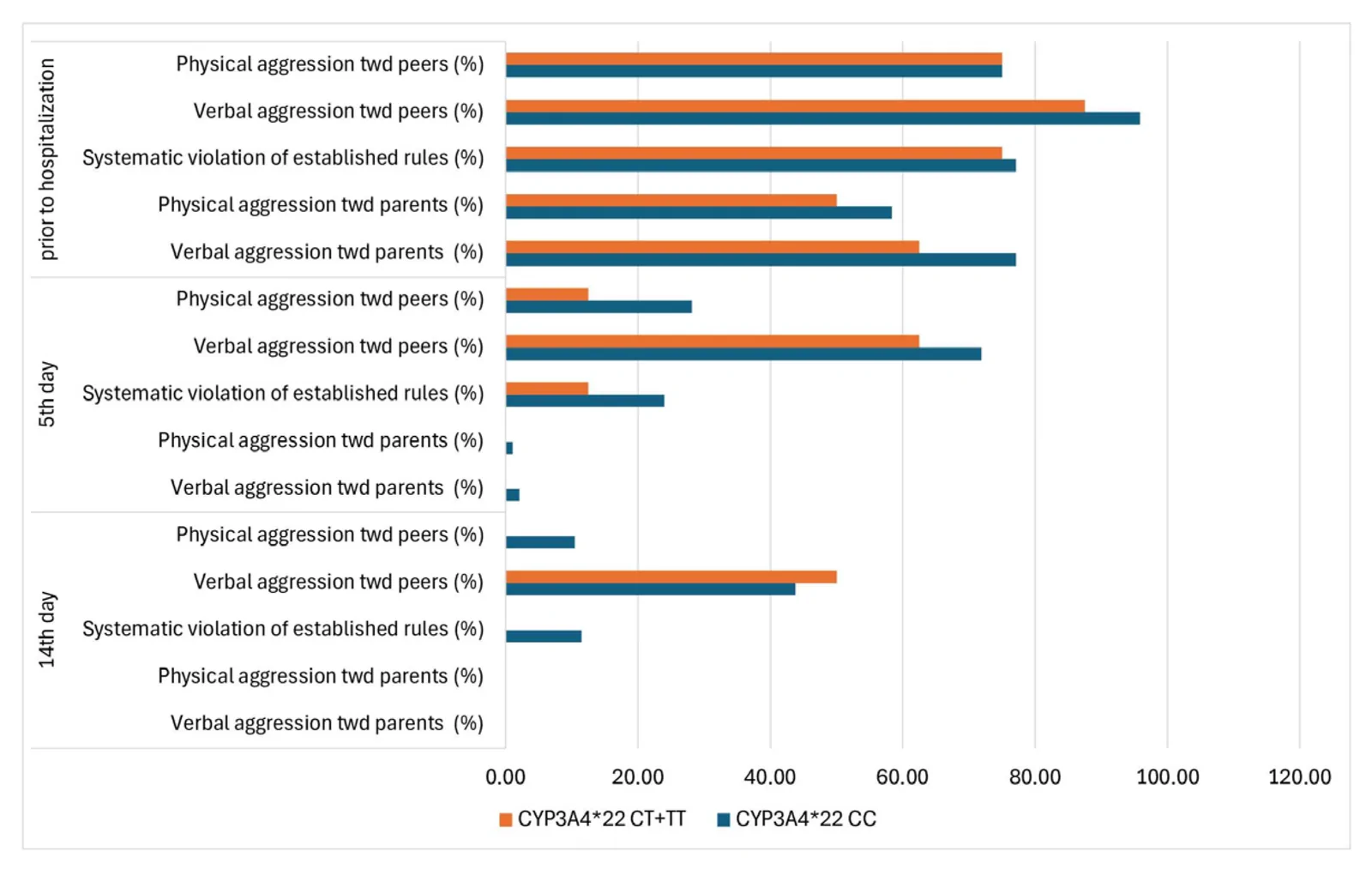
The prevalence of specific symptoms of conduct disorders according to CYP3A4*22 polymorphism status (CC vs. CT + TT).

**Figure 2:**
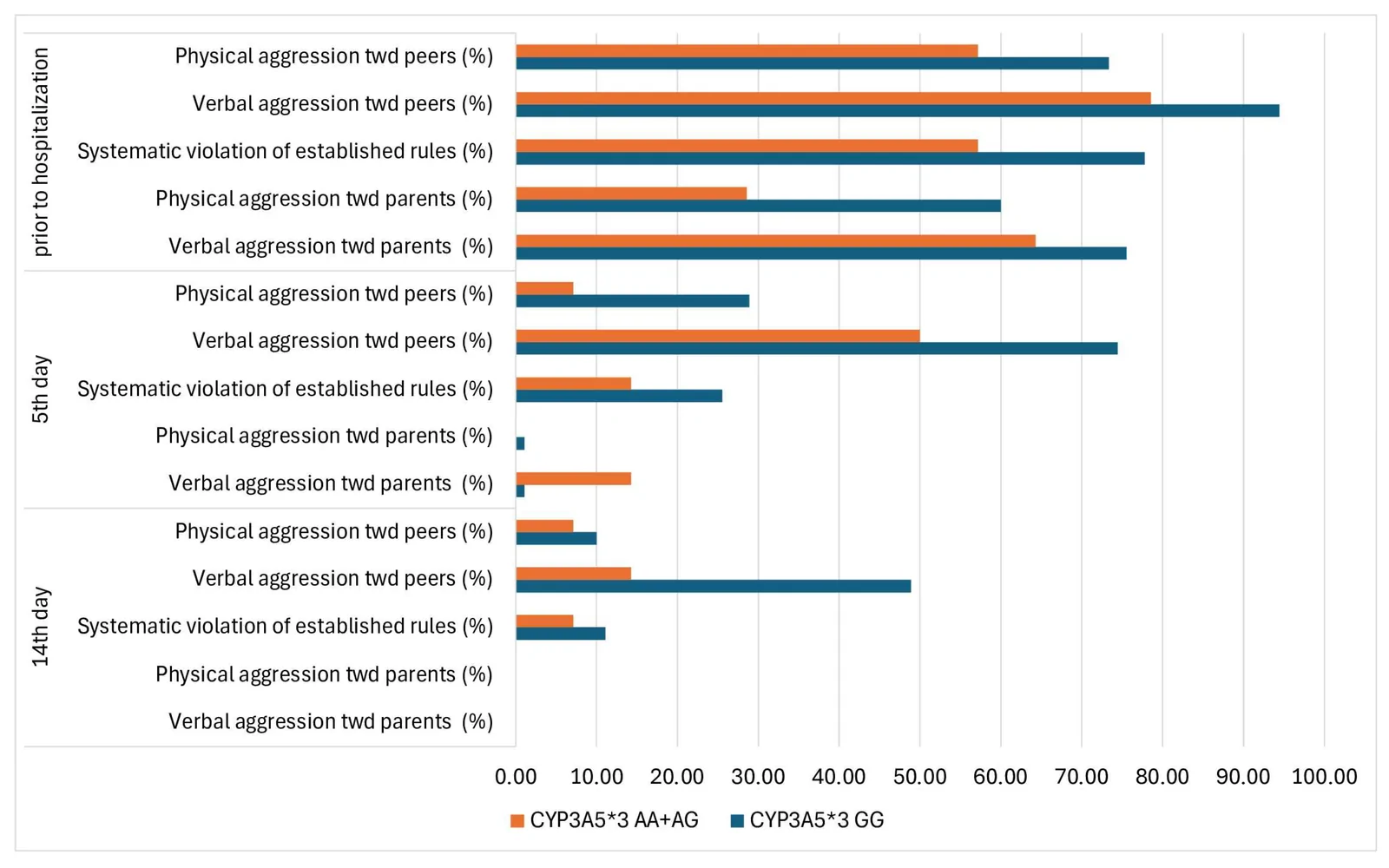
The prevalence of specific symptoms of conduct disorders according to CYP3A5*3 polymorphism status (TT vs. CC + CT).

An assessment of the severity of behavioral disorder symptoms based on CYP3A4*22 and CYP3A5*3 genotypes revealed no statistically significant differences (Figure 1 and Figure 2). In all patients, the severity of aggression and systematic violations of rules gradually decreased over time.

### 2.3. Analysis of Treatment Safety Based on CYP3A4*22 and CYP3A5*3 Genotypes

An analysis of the frequency of ADRs according to the UKU scale symptoms frequency revealed no statistically significant associations (Supplementary Table S4).

A comparison of complaint frequency regarding specific ADRs on day 5 revealed that CYP3A4*22 carriers reported asthenia or fatigue more often (37.5% vs. 9.4%; *p* = 0.017; after Yates’ correction: *p* = 0.044).

### 2.4. Analysis of Treatment Effectiveness Based on CYP2D6 Metabolism

**Figure 3:**
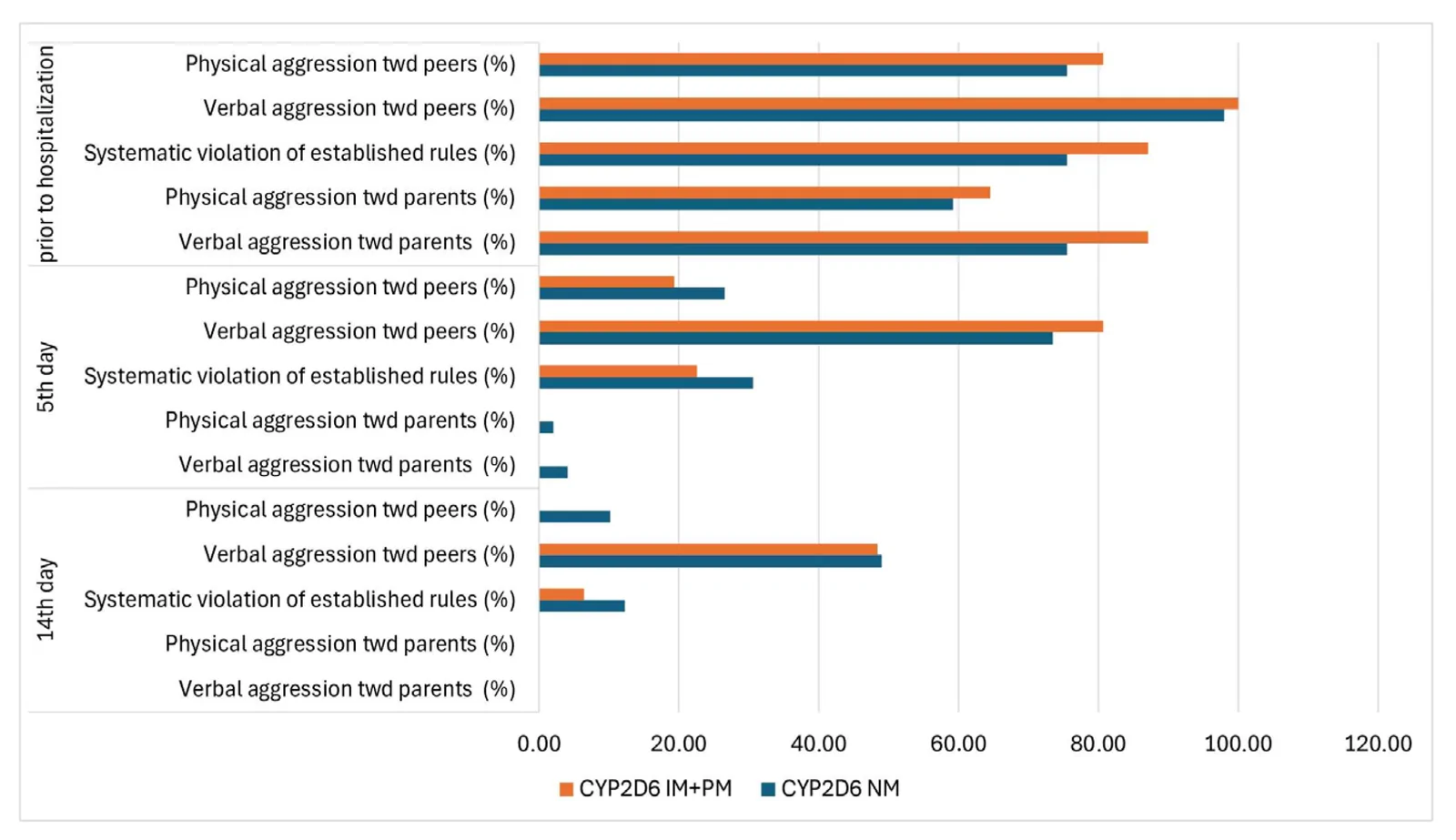
The prevalence of specific symptoms of conduct disorders according to *CYP2D6* metabolism rate. Notes: NM—normal metabolizer; IM—intermediate metabolizer; PM—poor metabolizer.

A comparison of CGI-S, CGI-I, and CGAS scores between patients with different CYP2D6 metabolic profiles did not reveal any significant associations, either for the overall sample or for the risperidone subgroup (Supplementary Table S3). Assessments of the severity of aggression and systematic rule-breaking also decreased significantly from the time of hospitalization through 14 days, with no significant differences based on CYP2D6 metabolic status (Figure 3). We do not present the results of the analysis for the overall sample, as the associations between CYP2D6 metabolism and risperidone are more significant.

### 2.5. Analysis of Treatment Safety Based on CYP2D6 Metabolism

A comparison of the frequency of ADR reports according to the UKU scale between CYP2D6 NM and IM + PM groups did not reveal any statistically significant associations (Supplementary Table S4). This finding held true for both the overall sample and the risperidone subgroup.

In the overall sample, no associations were found between the frequency of complaints about specific ADRs and CYP2D6 metabolism rate.

Analysis of the frequency of individual ADRs on day 5 revealed one significant association in the risperidone subgroup: patients with CYP2D6 IM + PM reported headaches more frequently (16.1% vs. 2%; *p* = 0.02; after Yates’ correction: *p* = 0.058). On day 14, no differences in the frequency of individual ADRs were observed.

## 3. Discussion

We identified associations between carrier status of the CYP3A4*22 and CYP3A5*3 polymorphisms, CYP2D6 metabolism, and the efficacy and safety of pharmacotherapy for conduct disorders in neurotypical boys. We included patients without a diagnosis of ASD or intellectual disability. We ruled out intellectual disability through clinical assessment; for some patients, Wechsler test results were also available. We compared Wechsler test scores to rule out their influence on the results.

A strength of our study is the duration of the observation period. We assessed the efficacy and safety of the treatment during the first two weeks. This is important for identifying patients at high risk of adverse drug reactions. Although we were unable to assess the longer-term effects of the treatment, we were able to examine in detail the changes observed on days 5 and 14 of treatment. Previous pharmacogenetic studies covered long follow-up periods, which meant it was not possible to focus solely on early adverse reactions. Meanwhile, designing a study to identify early outcomes allows for a more accurate identification of risk factors for treatment failure and poor tolerability, which is particularly important in pediatric practice.

The low prevalence of CYP3A4*22 and CYP3A5*3 makes it difficult to assess their associations with drug therapy outcomes. These polymorphisms are rare among Caucasians, but their impact on drug therapy outcomes may prove to be significant [7,25,40]. Our study identified 6.9% of carriers of the CYP3A4*22 polymorphism and 12.1% of carriers of the CYP3A5*1 polymorphism. These findings are consistent with previous studies conducted on other Russian patients [33,34,35]. This circumstance significantly reduced the sample size for statistical analysis; a similar problem was observed in a large Norwegian study of the pharmacokinetics of quetiapine [31]. The frequency of CYP3A5*1 is higher in the Chinese population, so research on this topic is more relevant [30]. The low prevalence of the CYP3A4*22 and CYP3A5*1 polymorphisms among Caucasians calls into question the potential clinical significance of genotyping for these variants.

In addition, we had to exclude from the analysis patients who were prescribed carbamazepine. Clinically significant induction of the CYP3A4 isoenzyme while taking carbamazepine usually requires an average of 7–14 days of treatment at a dose of at least 300 mg/day [41]. We cannot completely rule out the influence of carbamazepine, so we excluded these patients from our sample.

The safety analysis revealed only one association between reports of ADRs and the presence of the CYP3A4*22 polymorphism. The association of ADR with CYP3A4*22 is logical. Since this was an isolated finding, it should be verified in future studies with a larger sample size.

Previous studies examining the association between the CYP3A4*22 and CYP3A5*3 polymorphisms and the efficacy and safety of antipsychotics were based on a different patient population, which limits the comparability of our results. The studies by Shilbayeh et al. (2024), Hermans et al. (2023), and Kloosterboer et al. (2021) examined children with autism spectrum disorder [7,9,42]. The studies by Zhao et al. (2025) and Lin et al. (2024) report on adult patients with schizophrenia who were taking quetiapine [29,30].

Our study included various antipsychotics. Most patients received risperidone. In an Asian population, CYP3A5*3 carriers were associated with higher quetiapine concentrations compared to CYP3A5*1 carriers [29,30]. However, there is no significant association between the CYP3A4*22 and CYP3A5*3 polymorphisms and the serum concentrations of aripiprazole [9] or risperidone [7,42]. Risperidone is metabolized primarily by CYP2D6, but CYP3A4 and CYP3A5 also contribute to its metabolism [43]. To date, very few pharmacogenetic studies of *CYP3A4/CYP3A5* have been conducted in relation to antipsychotics. While these two genes are highly polymorphic, the known polymorphisms occur very infrequently. Studies of tacrolimus have demonstrated a significant role for CYP3A5*3 (rs776746) carrier status: the presence of this polymorphism requires a 50–100% dose reduction compared to CYP3A5*1 [19]. The role of CYP3A5*3 genotype in midazolam plasma concentrations has also been demonstrated [25], although there is evidence that CYP3A5*3 is less significant for midazolam than for tacrolimus [44].

The significance of CYP3A5*3 genotyping lies in the fact that, in the presence of the active allele A (designated CYP3A5*1), the expression of the CYP3A5 isoenzyme accounts for more than 50% of all cytochromes in the CYP3A subfamily [15]. In other words, in the presence of accelerated CYP3A5 metabolism, even a reduction in CYP3A4 metabolism loses its potential significance. According to the findings of Zhao et al. (2025) [30], carrying a single active allele A (CYP3A5*1) significantly reduces the serum concentration of quetiapine. This may affect the efficacy of treatment. Unfortunately, we cannot comment on long-term efficacy in our study. Regarding the short-term efficacy of antipsychotics, as well as safety, the CYP3A5*3 polymorphism was not associated with either efficacy or safety in our study. Of course, we understand that our patients took very little quetiapine. However, we can state that *CYP3A4* and *CYP3A5* genotyping has minimal prognostic value in the selection of antipsychotics that are partially metabolized by the relevant isoenzymes.

We found no significant association between CYP2D6 metabolism rates (NM vs. IM + PM) and the efficacy or safety of treatment. There were no significant differences among patients in terms of antipsychotic dosages or the frequency of ADR reports. The presence of only one significant association may indicate a Type I error and cannot serve as confirmation of the clinical significance of *CYP2D6* genotyping in children with behavioral disorders. We find it difficult to explain why our study did not yield significant results for CYP2D6, particularly when analyzing the subgroup of patients taking risperidone.

To date, pharmacogenetic studies of pharmacotherapy for behavioral disorders have been conducted only in children with autism spectrum disorder (ASD). These studies have primarily examined the associations between *CYP2D6* polymorphisms and the plasma concentrations of risperidone or aripiprazole [6,7,10,42,45]. Few studies have also examined adverse reactions or the effectiveness of treatment for conduct disorders [6,42,45]. Overall, to date, few pharmacogenetic studies have been conducted on the efficacy and safety of antipsychotics for conduct disorders in children with ASD [46,47]. The issue of mixed samples applies to both *CYP3A4/CYP3A5* and *CYP2D6*: some studies do not specify the patients’ diagnoses [6,45].

In our study, patients were taking various antipsychotics. Although we were able to identify a subset of patients who were prescribed only risperidone, we still consider the analysis of the overall sample to be significant. In the study by Varney et al. (2025) [48], patients with psychotic disorders, schizophrenia, and bipolar affective disorder were taking various antipsychotics. However, after pooling the results, the authors found significant associations with the metabolic rates of CYP3A4 and CYP2D6. Thus, our study also contributes to the understanding of the significance of pharmacogenetic predictors of the effectiveness and safety of antipsychotics.

Although we excluded patients taking carbamazepine, we did not exclude those prescribed fluvoxamine or amitriptyline from the analysis. These antidepressants are CYP2D6 substrates and may therefore affect the rate of metabolism. However, given the small number of cases (*n* = 3), we decided not to exclude these patients. Overall, likely phenoconversion of CYP3A4, CYP3A5, and CYP2D6 may contribute to the outcomes of psychopharmacotherapy. According to a study by Gerlach et al. (2025), clinically significant phenoconversion of cytochrome isoenzymes due to known modulators may affect up to 24% of patients [49]. This is the result of a review of the guidelines for personalized psychopharmacotherapy. On the other hand, it is impossible to completely avoid phenoconversion, so the best approach is to determine the isoenzyme metabolism rate in vivo [50]. However, this procedure is technically complex and not always available, so it is not used in routine practice.

An important limitation of our study is the short observation period. We were constrained by the length of the patient’s hospital stay. As a result, our study design did not allow us to assess long-term adverse reactions or to monitor the efficacy of antipsychotics in an outpatient setting. However, we focused on early adverse reactions to antipsychotics. The effectiveness of the treatment may be called into question because patients’ behavior could have changed due to being in unfamiliar surroundings. Consequently, we must allow for false-positive results. However, the results of the adverse reaction assessment should be trusted more than the assessment of treatment effectiveness.

Currently, the population of children with mental disorders has not been sufficiently studied in pharmacogenetic research. Existing recommendations for the personalized prescribing of psychotropic medications are based on studies of adult patients with schizophrenia and depressive episodes [1,3,51]. There have been numerous studies on the pharmacogenetics of antipsychotics in children with autism spectrum disorders; however, no guidelines have yet been developed for the personalized use of antipsychotics in this patient group [1,52]. It is interesting to note that very few studies in the literature have been devoted to the pharmacogenetics of antipsychotics in neurotypical children with conduct disorders [5,53]. Most studies involve mixed cohorts, among whom conduct disorders are cited as an indication for antipsychotic treatment [54,55]. In reality, we currently know very little about the actual significance of personalized prescribing of antipsychotics in this patient group.

Our study raises more questions than it answers, as we obtained unexpected negative results. We recognize that our study has many methodological shortcomings. However, the key issue is that, in real-world settings, no differences in the effectiveness and safety of antipsychotics were identified based on polymorphisms in *CYP3A4*, *CYP3A5*, and *CYP2D6*. The high clinical significance of these genes, demonstrated in previous studies of adults and children, has not been confirmed in a sufficiently large sample of children with conduct disorders. We must consider whether pharmacogenetic testing is even necessary at all when selecting an antipsychotic for neurotypical children with conduct disorders. According to our findings, genotyping for *CYP3A4*, *CYP3A5* and *CYP2D6* has no prognostic value for early clinical effectiveness or early adverse drug reactions to antipsychotics in children with conduct disorders. It can be assumed that drug dosages in this population are low. However, with slowed metabolism of isoenzymes, even starting doses increase the risk of early adverse reactions, which patients would report by the fifth day of observation.

It is possible that other factors are responsible for the negative results. We did not assess the role of patient age in our study. Currently, there is evidence that a child’s age is important for the safety of psychotropic medications. Children under the age of 13 are less likely to report ADRs from antipsychotics. In a previous study, we observed varying frequencies of ADRs in children and adolescents with an acute psychotic episode [56]; this has also been confirmed in other studies [57,58,59]. On the other hand, there are studies indicating that children tolerate SSRIs worse compared to adolescents [60]. Few studies have evaluated the significance of CYP3A4, CYP3A5, and CYP2D6 metabolic rates in relation to a child’s age. In a study by T’jollyn et al. (2015), it was shown that *CYP2D6* genetic testing is a poor predictor of tramadol blood concentrations in children aged 2 to 13 years [61].

It follows that children aged 7 to 12 constitute a distinct group and require separate consideration in pharmacogenetic studies. One possible explanation is communication: children are less likely to report their ADRs compared to adolescents [57]. In this study, we were unable to compare children and adolescents. However, in the future, we plan to expand our sample size to clarify the role of age in pharmacogenetic studies of antipsychotics in neurotypical children with conduct disorders.

Limitations. Our study is a single-center, observational study, which imposes certain limitations on the generalizability of the results. Only boys were included; while this allowed us to avoid the influence of gender on the activity of the CYP3A4 and CYP3A5 isoenzymes, it also limited the generalizability of our results to girls. The short follow-up period did not allow for an assessment of long-term ADRs or the efficacy of antipsychotics. There were very few carriers of the CYP3A4*22 and CYP3A5*1 polymorphisms in our sample, which made it impossible to compare subgroups and increased the likelihood of Type I and II errors. We did not have a technical opportunity to genotype CYP2D6*1 duplications, so we did not determine “ultrarapid” metabolizers. Blood concentrations of antipsychotics were not measured in the study; we relied exclusively on clinical assessment of efficacy and safety. Patients received different antipsychotics, which we adjusted by converting dosages to chlorpromazine equivalents. Our study did not *include in vivo* phenotyping of CYP3A4, CYP3A5, and CYP2D6 isoenzyme activity; therefore, we did not account for potential phenoconversion.

## 4. Materials and Methods

### 4.1. Study Sample

The study was designed as an observational cohort study in naturalistic conditions. The study protocol was developed in accordance with the World Health Organization’s Declaration of Helsinki and approved at a meeting of the Local Ethics Committee of the Scientific-Practical Children’s and Adolescents Mental Health Center n.a. G.E. Sukhareva (Protocol No. 3/23 dated 9 November 2023).

Written informed consent was obtained from the individuals and their legal guardians for the publication of any potentially identifiable data included in this article.

The study included children who were hospitalized for behavioral disorders between 1 December 2023 and 30 November 2024. A total of 115 boys were included in the study.

Inclusion criteria:

- Children aged 7 to 12 years inclusive;
- Male gender;
- Presence of conduct disorders that lead to significant maladjustment and require pharmacotherapy;
- No contraindications to the use of antipsychotics;
- Consent to participate in the study from the participant and the child’s legal guardians.

Exclusion criteria:

- Intellectual disability confirmed by the Wechsler test (IQ < 70);
- Presence of a schizophrenia spectrum disorder;
- Presence of a comorbid anxiety disorder;
- Presence of comorbid depressive disorder;
- Presence of contraindications to pharmacotherapy prescribed by a physician for the treatment of conduct disorders;
- Refusal to participate in the study.

Patients were enrolled in the study 1–2 days after admission, prior to the initiation of antipsychotic treatment. Key clinical and demographic data—including age, number of previous hospitalizations, and age at onset of behavioral disturbances—were extracted from the patients’ medical records.

At the time of the patient’s enrollment in the study, a clinical interview was conducted, including standardized scales to assess the severity of the patient’s mental disorder. Subsequently, these same scales were used to evaluate the effectiveness of pharmacotherapy.

The list of scales used to assess the patient’s mental state:

- Child Global Assessment Scale (CGAS) [62]. The higher the CGAS score, the better the patient’s level of adjustment.
- Clinical Global Impression—Severity (CGI-S) [63]. The higher the score, the greater the severity of the mental disorder.
- Clinical Global Impression—Improvement (CGI-S) [63]. The lower the score, the more pronounced the patient’s improvement.
- The Conners Scale [64]—completed by the patient’s parents. The scale assesses the severity of attention deficit and hyperactivity in the patient.
- Clinical assessment of conduct disorders using a set of parameters. For each item, the researcher had to answer “yes” or “no.” The set includes the following parameters:
  ○ verbal aggression toward parents,
  ○ verbal aggression toward peers,
  ○ physical aggression toward parents,
  ○ physical aggression toward peers,
  ○ repeated violations of established rules.

This was not a rating scale; rather, it consisted of individual symptoms, each of which served as a binary categorical variable for analysis.

The list of scales used to monitor the safety of drug therapy:

- UKU Side Effects Rating Scale (UKU SERS) [65]. We used the scale as a checklist, noting only the presence or absence of symptoms, without taking into account the severity of the ADR. This scale includes four subscales: “Mental Disorders,” “Neurological Disorders,” “Autonomic Nervous System Disorders,” and “Other Disorders”. However, we were unable to calculate the UKU SERS score because we assessed only the number of ADR reports. This was due to a limitation of the sample: it was difficult for children aged 7–12 to assess the severity of ADRs, and we sought to avoid misleading results.
- The Simpson-Angus Scale for Assessing Extrapyramidal Adverse Reactions (SAS) [66]. The result is a total score that determines the severity of the patient’s extrapyramidal symptoms.

### 4.2. Analysis of Pharmacotherapy

The researcher had no influence over the prescribing of psychopharmacotherapy by the attending physician. All psychotropic medications received by the patient were recorded in an individual medical record. The daily doses of medications received at the time of study enrollment, as well as on days 5 and 14 of observation, were recorded. Antipsychotic doses were converted to chlorpromazine equivalents to standardize further analysis [67].

All patients received an antipsychotic as their primary treatment. In some cases, an antidepressant, a mood stabilizer, or an anticholinergic agent (biperiden) was added. Such cases were classified as polypharmacy and were taken into account in the analysis.

Since carbamazepine can induce the activity of CYP3A4/5 isoenzymes [41], we identified patients who had been prescribed carbamazepine. We excluded these patients from the analysis of the association between carrier status of the CYP3A4*22 and CYP3A5*3 polymorphisms and the efficacy and safety of pharmacotherapy.

### 4.3. Genotyping

On the day of enrollment in the study, 5 mL of blood was collected from each patient into disposable sterile EDTA vacuum tubes for subsequent genotyping. The collection of biological material was performed concurrently with routine laboratory tests and did not require additional venipunctures. The blood was frozen at −20 °C, transported to the laboratory, and subsequently stored at −70 °C.

DNA extraction and genotyping of the samples were performed as they were received.

DNA extraction from venous blood was performed using the column-based method with the QIAamp DNA Blood Mini Kit (Qiagen, Hilden, Germany). Concentration determination and quality assessment of the obtained DNA preparations were performed using a Qubit 4 fluorometer (Thermo Fisher Scientific, Waltham, MA, USA) and a NanoDrop ND-1000 spectrophotometer (Thermo Fisher Scientific, Waltham, MA, USA).

Prescence of CYP3A4*22 rs35599367 (C>T), and CYP3A5*3 (rs776746, 6986T>C), CYP2D6*3 (rs35742686), CYP2D6*4 (G1846A, rs3892097), CYP2D6*6 (rs5030655), CYP2D6*10 (C100T, rs1065852), CYP2D6*41 (rs28371725) was determined by real-time polymerase chain reaction (PCR) using commercial reagent kits and the CFX96 TouchTM Real-Time PCR Detection System (Bio-Rad, Hercules, CA, USA). Commercially available kits manufactured by “Sintol” (Moscow, Russia) were used for amplification.

All patients were divided into subgroups based on the genotypes of CYP3A4*22 or CYP3A5*3 polymorphism. Based on the CYP3A4*22 polymorphism, they were divided into two subgroups: CC and CT + TT. Due to the high prevalence of the CYP3A5*3 polymorphism (T allele), we identified subgroups of homozygous carriers of CYP3A5*3 (TT) and carriers of the active CYP3A5*1 allele (CT genotypes). Subsequently, the subgroups were compared to identify associations with patients’ clinical parameters.

CYP2D6 metabolism rates were determined in accordance with the recommendations of the DPWG [1]. Based on the analysis of *CYP2D6* gene polymorphisms, patients were divided into two groups: NM (normal metabolizers) and IM + PM (intermediate and poor metabolizers). We grouped PM and IM together for analysis, as the subgroup of PM patients in our sample was too small for such a comparison to have sufficient statistical power.

The selection of polymorphisms was based on their high significance for the rate of substrate metabolism by CYP isoenzymes [10,20,30,68].

### 4.4. Statistical Analysis of the Results

Statistical analysis was performed using SPSS Statistics (version 26.0, IBM Corp., Armonk, NY, USA). The preliminary sample size was based on the number of patients in previous studies on pharmacogenetic risk factors for adverse reactions to antipsychotics [7,9,10]. Due to the non-normal distribution of the data, nonparametric tests were used to compare quantitative variables between groups. The results of the quantitative variables are presented as the median and quartiles—Me [Q1; Q3].

The Mann-Whitney U test was used to compare the identified subgroups at a single time point based on quantitative variables. The frequencies of categorical variables were compared using Pearson’s chi-square test; for 2 × 2 comparisons, Fisher’s exact test was used. Bonferroni correction was applied to adjust for multiple comparisons. The calculation of the Hardy-Weinberg equilibrium of genotype distributions was performed using an online calculator [69].

**Figure 4:**
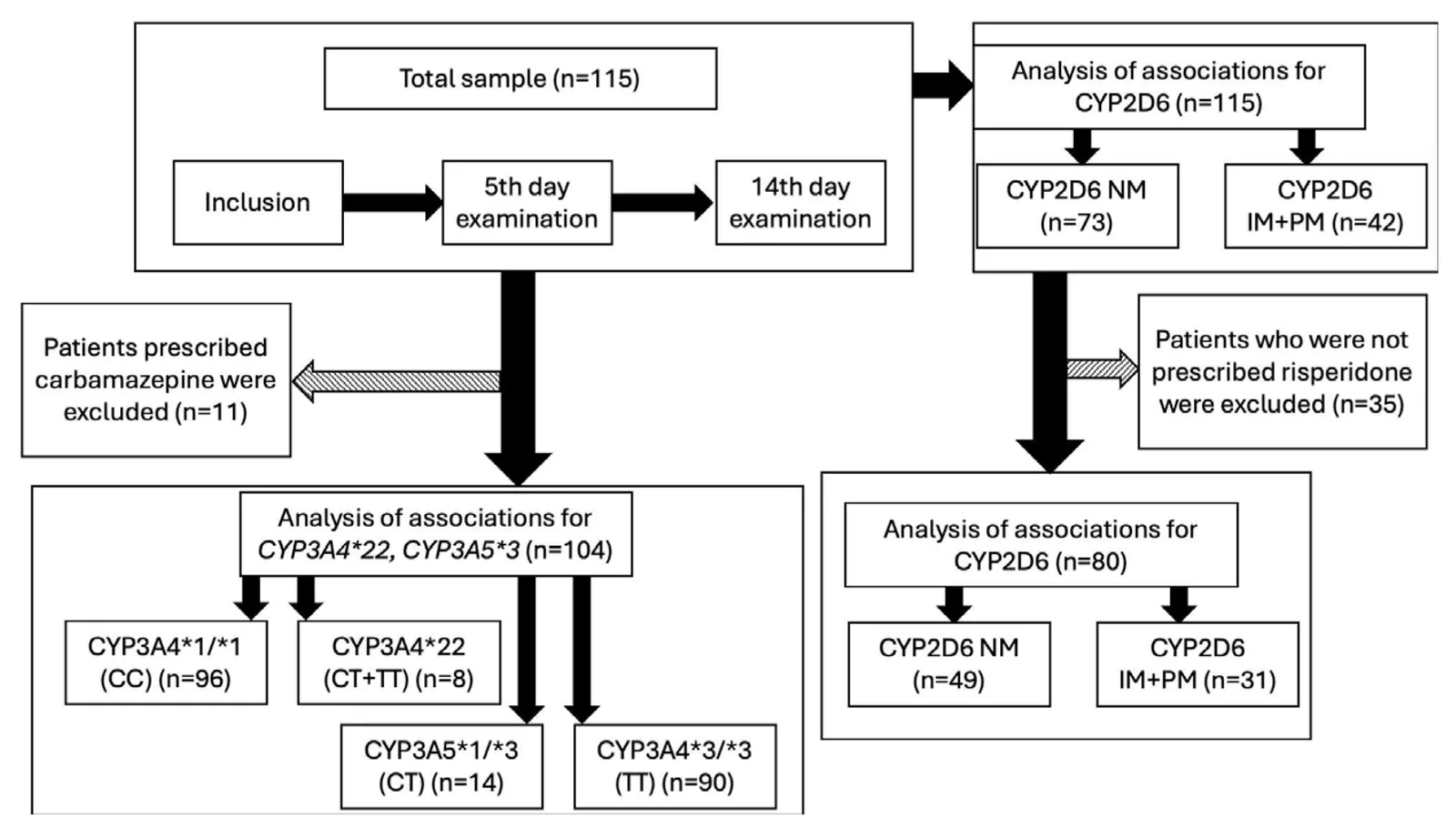
Flowchart for analyzing associations between the effectiveness and safety parameters of antipsychotics and polymorphisms CYP3A4*22, CYP3A5*3, *CYP2D6*.

The patient cohort was analyzed for factors requiring exclusion of patients. To analyze associations between treatment outcomes and CYP3A4*22 and CYP3A5*3 polymorphisms, patients prescribed carbamazepine were excluded from the analysis. Since risperidone was the most frequently prescribed medication in our sample, the analysis of associations with the CYP2D6 phenotype was conducted in two stages—first for the entire sample (*n* = 115), then for the risperidone subgroup (*n* = 80). A schematic representation of the analysis of our sample is shown in Figure 4.

When analyzing the data, the influence of patients’ demographic and clinical characteristics on the outcomes under study—including the effect of polypharmacy—was taken into account. Separate pairwise comparisons were conducted between subgroups defined by genotypes of the CYP3A4*22 and CYP3A5*3 polymorphisms and the CYP2D6 predicted phenotype, focusing on the frequency of additional pharmacotherapy, antipsychotic doses, and patient demographic characteristics.

## 5. Conclusions

Pharmacogenetic testing for *CYP3A4/CYP3A5* has not been sufficiently studied for the prescription of antipsychotics. Our study identified a few statistically significant associations between CYP3A4*22 (rs35599367), *CYP2D6*, and safety of antipsychotics among neurotypically developed boys with conduct disorders. To date, no guidelines have been developed for the personalized selection of treatment for conduct disorders in childhood.

Our findings cast serious doubt on the rationale for genotyping CYP3A4*22 (rs35599367) and CYP3A5*3 (rs776746) to predict the early efficacy and safety of antipsychotics in children with conduct disorders. The same is true for genotyping *CYP2D6* gene polymorphisms, as their clinical significance for these study conditions is also called into question. However, further research of this kind is needed to determine the implications of *CYP3A4*, *CYP3A5*, and *CYP2D6* genotyping in neurotypical children undergoing pharmacotherapy for conduct disorders. The negative results we obtained are valid only for the short 14-day observation period.

## Figures and Tables

**Table 1:** Baseline characteristics of the overall sample, subsamples for analysis of associations based on CYP3A4*22, CYP3A5*3 carrier status (*n* = 104), CYP2D6 phenotype in those taking risperidone (*n* = 80).

Variables	All (*n* = 115)	Subsample for CYP3A4/5 (*n* = 104)	Subsample for risperidone and CYP2D6 (*n* = 80)
Age, years	10 [9;11]	10 [9;11]	10 [9;11]
Height, santimeters	143 [137;149]	144 [137;148]	144 [137;149]
Body weight, kg	37 [31.5;43]	37 [31;42]	37 [32;42]
Body mass index	17.9 [16.1;20.8]	17.9 [16.1;20.5]	17.6 [16.1;20.3]
Age at onset of conduct disorder symptoms (years)	9 [8;10]	9 [8;10]	9 [8;10]
Total number of hospitalizations (including this one)	1 [1;1]	1 [1;1]	1 [1;1]
Duration of conduct disorders prior to study enrollment (months)	2 [2;5]	2 [2;4]	2 [2;4]
Wechsler Test, total score (*n* = 77)	89 [80;97]	89 [79;99]	89 [82;97]
Connors Test, total score	29 [24;36]	29 [25;37]	29 [25;37]

**Table 2:** Parameters of pharmacotherapy administered to patients during the observation period. Analysis of pharmacotherapy parameters according to CYP3A4*22, CYP3A5*3 and CYP2D6 status.

Variables	All (*n* = 115)	CYP3A4*22		*p*	CYP3A5*3		*p*	CYP2D6		*p*
		CC (*n* = 96)	CT + TT (*n* = 8)		GG (*n* = 90)	AA + AG (*n* = 14)		NM (*n* = 49)	IM + PM (*n* = 31)	
Antipsychotic dose for days 1–2, mg/day	50 [50;100]	50 [50;104]	50 [50;50]	0.751	50 [50;108]	55 [50;96]	0.731	0.5 [0.5;1]	0.75 [0.5;1]	0.695
Antipsychotic dose on day 5, mg/day	100 [96;180]	100 [98;200]	100 [75;125]	0.424	100 [100;200]	100 [60;200]	0.398	1 [1;1.75]	1 [1;2]	0.854
Antipsychotic dose on day 14, mg/day	150 [100;200]	150 [100;200]	150 [100;175]	0.475	150 [100;200]	150 [100;200]	0.724	2 [1;2]	1.5 [1;2]	0.145
Antipsychotic	-	-	-	-	-	-	-	-	-	-
Risperidone	80 (69.6%)	68.8%	100%	>0.9	72.2%	64.3%	>0.9	100%	100%	-
Haloperidol	3 (2.6%)	3.1%	0	>0.9	3%	0	>0.9	-	-	-
Aripiprazole	7 (6.1%)	7.3%	0	>0.9	6.7%	7.1%	>0.9	-	-	-
Periciazine	21 (18.3%)	16.70%	0	>0.5	13.3%	28.6%	>0.5	-	-	-
Thioridazine	1 (0.9%)	1%	0	>0.9	1.1%	0	>0.9	-	-	-
Chlorpromazine	2(1.7%)	2.1%	0	>0.9	2.2%	0	>0.9	-	-	-
Levomepromazine	1 (0.9%)	1%	0	>0.9	1.1%	0	>0.9	-	-	-
Mood stabilizer	14 (12.17%)	2 (2.1%)	1 (12.5%)	0.2154	2 (2.2%)	1 (7.1%)	0.3549	5 (10.2%)	2 (6.5%)	0.7002
Antidepressant	8 (6.96%)	5 (5.2%)	2 (25%)	0.142	7 (7.8%)	0 (0%)	0.5897	3 (6.1%)	3 (9.7%)	0.6721
Anticholinergic drug	26 (22.61%)	24 (25.0%)	0 (0%)	0.1931	22 (24.4%)	2 (14.3%)	0.5133	8 (16.3%)	3 (9.7%)	0.5153

## Data Availability

The original contributions presented in this study are included in the article/Supplementary Material. Further inquiries can be directed to the corresponding author.

## Supplementary Materials

The following supporting information can be downloaded at: https://www.mdpi.com/article/10.3390/ph19091401/s1, Table S1: Baseline characteristics of subsample for analysis of *CYP3A4/CYP3A5*; Table S2: Baseline characteristics of patients according to CYP2D6 metabolism rate; Table S3: Analysis of the effectiveness of pharmacotherapy based on psychometric scales; Table S4: Results of the pharmacotherapy safety analysis according to the UKU SERS and SAS scales.
