# Supplementary material for: Genetic Polymorphisms CYP3A4*22, CYP3A5*3, and CYP2D6 Predicted Phenotypes Are Not Associated with Antipsychotic Treatment Outcomes in Neurotypical Prepubertal Boys with Conduct Disorders
Source: Pharmaceuticals (Basel). 2026 Sep 4;19(9):1401. doi: 10.3390/ph19091401 (PMC13610822; doi:10.3390/ph19091401)
Supplement: Supplementary file 1 [file pharmaceuticals-19-01401-s001.zip › pharmaceuticals-4510239-supplementary.pdf]

Supplementary Material

Supplement Table S1. Baseline characteristics of subsample for analysis of *CYP3A4/CYP3A5.* Patients were divided related to *CYP3A4*22* or *CYP3A5*3* polymorphisms

Variables	CYP3A4*22		p	CYP3A5*3		p
	CC (n=96)	CT+TT (n=8)		TT (n=90)	CT+CC (n=14)	
Age, years	10[9;11]	10[9,5;11]	0,585	10[9;11]	10[9;12]	0,518
Height, santimeters	142[135,5;148]	144,5[138;147,5]	0,671	142,5[136;148]	143,5[134;150]	0,788
Body weight, kg	37[30,5;42,5]	37,5[33,5;43]	0,900	37[31,5;44]	37,5[26;41]	0,470
Body mass index	17,95{16,1;20,9]	17,45[15,65;20,55]	0,788	18,1[16,2;21,1]	17,1[15,1;18,9]	0,074
Age at onset of conduct disorder symptoms (years)	9[8;10]	9,5[7,5;10]	0,957	9[8;10]	9,5[9;10]	0,470
Total number of hospitalizations (including this one)	1[1;1,5]	1[1;1]	0,521	1[1;1]	1[1;2]	0,436
Wechsler Test, total score (n=77)	88,5[79,5;98]	92,5[77;107]	0,630	85[79;97]	94[87;107]	0,135
Conners Scale, total score	29[25;36]	32,5[25,5;39,5]	0,679	29[25;37]	28,5[24;35]	0,500

Supplement Table S2. Baseline characteristics of patients according to CYP2D6 metabolism rate. A separate comparison was conducted for the subset of patients who were prescribed risperidone only

Variables	CYP2D6		p	CYP2D6 (only risperidone)		p
	NM (n=73)	IM+PM (n=42)		NM (n=49)	IM+PM (n=31)	
Age, years	10 [9; 11]	9 [9;11]	0,082	10[9;11]	9[8;10]	0,048
Height, santimeters	144[138;150]	141 [135;146]	0,135	145[138;150]	142[134;145]	0,045
Body weight, kg	39[32;44]	35,5 [30;40]	0,101	39[33;44]	35[30;40]	0,060
Body mass index	18,4[16,5;21,1]	17,2 [15,6;19,8]	0,143	18,3[16,8;20,9]	17,1[15,6;19,8]	0,095
Age at onset of conduct disorder symptoms (years)	9[8;11]	9[7;10]	0,344	9[8;10]	9[7;10]	0,302
Total number of hospitalizations (including this one)	1[1;1]	1[1;2]	0,293	1[1;1]	1[1;1]	0,687
Wechsler Test, total score (n=77)	91[80;101]	87[79;94]	0,581	91[82;97]	89[83;96]	0,700
Conners Scale, total score	29[25;36]	28,5[24;36]	0,493	29[25;37]	29[27;36]	0,945

Supplement Table S3. Analysis of the effectiveness of pharmacotherapy based on psychometric scales. *Notes: CGAS - Child Global Assessment Scale; CGI-S - Clinical Global Impression – Severity; CGI-I - Clinical Global Impression – Improvement*

Variables	CYP3A4*22		p	CYP3A5*3		p	CYP2D6 (risperidone only)		p
	CC (n=96)	CT+TT (n=8)		GG (n=90)	AA+AG (n=14)		NM (n=49)	IM+PM (n=31)	
CGAS score at 1 day	6[5;7]	6,5[5,5;7]	0,400	6[5;7]	6[5;6]	0,458	6[5;7]	6[5;7]	0,865
CGAS score on day 5	7[6;7]	7[7;7]	0,261	7[6;7]	6,5[6;7]	0,378	7[6;7]	7[6;7]	0,890
CGAS score on day 14	7[6;7]	7[7;7]	0,678	7[6,5;7]	7[6;7]	0,924	7[6;7]	7[6,5;7]	0,659
CGI-S score at 1 day	4[3;4]	4[3;4]	0,710	4[3;4]	4[3;4]	0,831	4[3;4]	3[3;4]	0,389
CGI-S score on day 5	3[3;3]	3[2,5;3]	0,234	3[3;3]	3[3;3]	0,823	3[3;3]	3[3;3]	0,333
CGI-S score on day 14	3[2;3]	3[2;3]	0,432	3[2;3]	3[2;3]	0,821	3[3;3]	3[2;3]	0,417
CGI-I score on day 5	3[3;4]	3[3;3]	0,215	3[3;4]	3[3;4]	0,862	3[3;4]	3[3;4]	0,425
CGI-I score on day 14	3[3;3]	3[3;3]	0,455	3[3;3]	3[3;3]	0,829	3[3;3]	3[3;3]	0,575

Supplement Table S4. Results of the pharmacotherapy safety analysis according to the UKU SERS and SAS scales.

Notes: ADR – adverse grug reaction; UKU SERS - UKU Side Effects Rating Scale; SAS - Simpson-Angus Scale. The UKU SERS scale was used as a questionnaire to assess adverse drug reactions, and the number of complaints for each subscale was taken into account.

Variables	CYP3A4*22		p	CYP3A5*3		p	CYP2D6 (risperidone only)		p
	CC (n=96)	CT+TT (n=8)		GG (n=90)	AA+AG (n=14)		NM (n=49)	IM+PM (n=31)	
UKU “Mental Disorders” subscale on day 5, total frequency	0 [0; 1]	0,5[0;1,5]	0,216	0[0;1]	0[0;0]	0,357	0[0;0]	0[0;1]	0,898
UKU “Neurological Disorders” subscale on day 5, total frequency	0[0;0]	0[0;0]	0,602	0[0;0]	0[0;0]	0,803	0[0;0]	0[0;0]	0,953
UKU Subscale “Autonomic Nervous System Disorders” on Day 5, total frequency	0[0;0]	0[0;0]	0,715	0[0;0]	0[0;0]	0,447	0[0;0]	0[0;1]	0,208
UKU “Other HR” subscale on day 5, total frequency	0[0;0]	0[0;0]	0,891	0[0;0]	0[0;0]	0,869	0[0;0]	0[0;0]	0,371
ADRs total frequency on day 5	0[0;2]	0[0;2]	0,370	0[0;2]	0[0;1]	0,292	0[0;1]	0[0;1]	0,145
SAS Scale on Day 5, total score	0[0;0]	0[0;0]	0,602	0[0;0]	0[0;0]	0,453	0[0;0]	0[0;0]	0,432
UKU “Mental Disorders” subscale on day 14, total frequency	0[0;0]	0[0;0,5]	0,501	0[0;0]	0[0;0]	0,466	0[0;0]	0[0;0]	0,744

UKU “Neurological Disorders” subscale on day 14, total frequency	0[0;0;]	0[0;0;]	0,786	0[0;0]	0[0;0]	0,644	0[0;0]	0[0;0]	0,767
UKU Subscale “Autonomic Nervous System Disorders” on Day 14, total frequency	0[0;0]	0[0;0]	0,889	0[0;0]	0[0;0]	0,508	0[0;0]	0[0;0]	0,932
UKU “Other Health Problems” subscale for day 14, total frequency	0[0;0]	0[0;0]	0,814	0[0;0]	0[0;0]	0,597	0[0;0]	0[0;0]	0,853
ADRs total frequency on day 14	0[0;1]	0[0;1]	0,573	0[0;1]	0[0;0]	0,172	0[0;0]	0[0;1]	0,577
SAS Scale on Day 14, total score	0[0;0]	0[0;0]	0,767	0[0;0]	0[0;0]	0,644	0[0;0]	0[0;0]	0,293
